# Supplementary material for: Pars Opercularis Underlies Efferent Predictions and Successful Auditory Feedback Processing in Speech: Evidence From Left-Hemisphere Stroke
Source: Neurobiol Lang (Camb). 2024 Jun 3;5(2):454–83. doi: 10.1162/nol_a_00139 (PMC11192514; doi:10.1162/nol_a_00139)
Supplement: Supplementary file 1 [file nol-5-2-454-s001.pdf]

Supplementary Materials

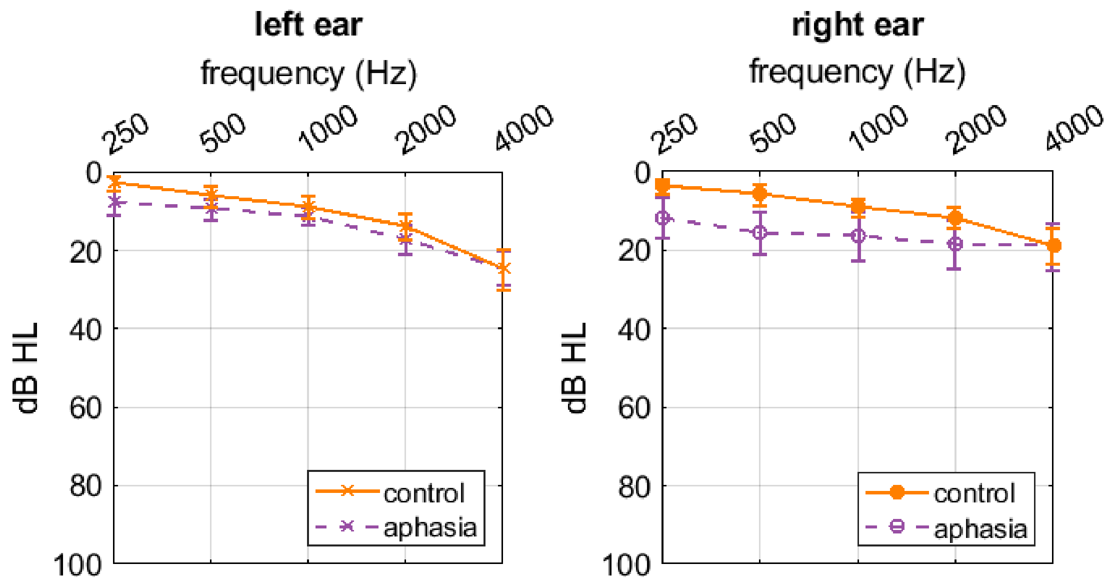

Figure S1. Audiograms for left and right ears. Hearing thresholds did not differ between controls and persons with aphasia at any frequency. Error bars represent the standard error.

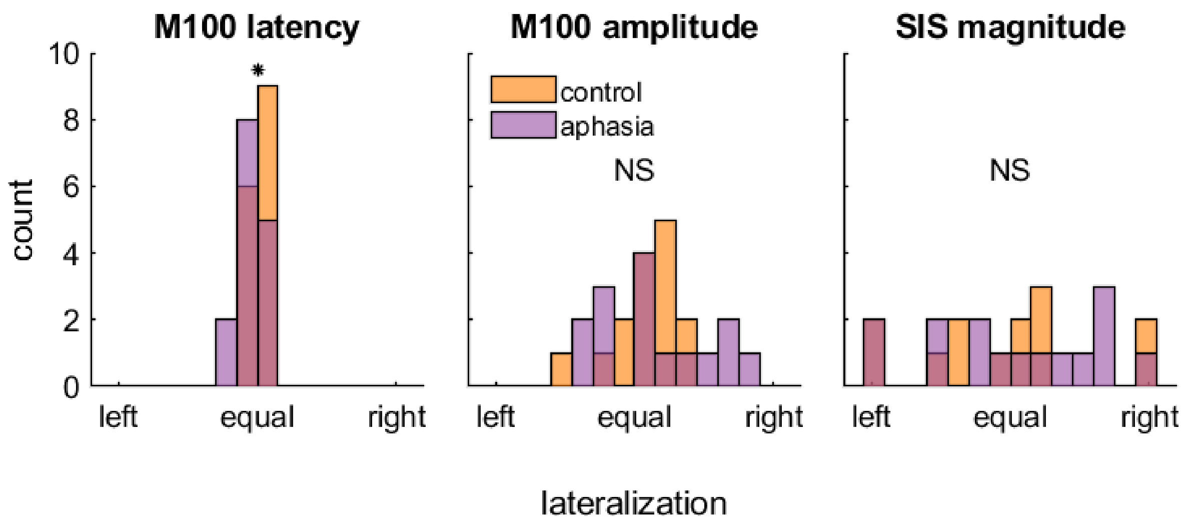

Figure S2. Laterality of neural responses. Each panel is a histogram depicting control (orange) and aphasic (purple) participants' laterality index for a given neural metric. Each laterality index is calculated as (right − left/(right + left), giving a range of values between fully left-lateralized and fully right-lateralized. **(A)** The laterality of listen-evoked M100 latencies significantly differed between control and aphasia, due to longer latencies in some lesioned left hemispheres. **(B)** The laterality of listen-evoked M100 amplitudes did not differ between the groups. **(C)** The laterality of the magnitude of speaking-induced suppression (SIS) did not differ between the groups. *Statistical significance:* * $p < 0.05$.
